# Unveiling the role of RAC3 in the growth and invasion of cisplatin‐resistant bladder cancer cells

**DOI:** 10.1111/jcmm.18473

**Published:** 2024-06-07

**Authors:** Haodong Li, Hongxuan Ma, JianHua Ma, Fei Qin, Siqi Fan, Shaopeng Kong, Sitao Zhao, Jianguo Ma

**Affiliations:** ^1^ Department of Urology Hebei Medical University Third Hospital Shijiazhuang China; ^2^ Faculty of Health and Behavioural Sciences The University of Queensland Queensland Australia; ^3^ Geriatrics Department Hebei Chengde Central Hospital Chengde China

**Keywords:** bladder cancer, cisplatin resistance, invasion, JNK, proliferation, RAC3

## Abstract

Bladder cancer is one of the most prevalent cancers worldwide, and its morbidity and mortality rates have been increasing over the years. However, how RAC family small GTPase 3 (RAC3) affects the proliferation, migration and invasion of cisplatin‐resistant bladder cancer cells remains unclear. Bioinformatics techniques were used to investigate the expression of RAC3 in bladder cancer tissues. Influences of RAC3 in the grade, stage, distant metastasis, and survival rate of bladder cancer were also examined. Analysis of the relationship between RAC3 expression and the immune microenvironment (TIME), genomic mutations, and stemness index. In normal bladder cancer cells (T24, 5637, and BIU‐87) and cisplatin‐resistant bladder cancer cells (BIU‐87‐DDP), the expression of RAC3 was detected separately with Western blotting. Plasmid transfection was used to overexpress or silence the expression of RAC3 in bladder cancer cells resistant to cisplatin (BIU‐87‐DDP). By adding activators and inhibitors, the activities of the JNK/MAPK signalling pathway were altered. Cell viability, invasion, and its level of apoptosis were measured in vitro using CCK‐8, transwell, and flow cytometry. The bioinformatics analyses found RAC3 levels were elevated in bladder cancer tissues and were associated with a poor prognosis in bladder cancer. RAC3 in BIU‐87‐DDP cells expressed a higher level than normal bladder cancer cells. RAC3 overexpression promoted BIU‐87‐DDP proliferation. The growth of BIU‐87‐DDP cells slowed after the knockdown of RAC3, and RAC3 may have had an impact on the activation of the JNK/MAPK pathway.

## 1 INTRODUCTION

Bladder cancer is among the most common malignancies globally. With over 573,000 new cases diagnosed and over 213,000 fatalities annually, the numbers are still continually increasing.^1^, ^2^ Muscle invasive and metastasis occur in approximately 25% of patients with bladder cancer.^3^ Cisplatin‐based neoadjuvant chemotherapy (NAC) improves the survival rate for patients with muscle invasive bladder cancers who undergo radical cystectomies.^4^ However, only 35% of patients with metastatic bladder cancer react to cisplatin treatment at first. Most patients with pre‐sensitive bladder cancer eventually develop resistance, which leads to poor patient prognoses allowing bladder cancer cells to continuously proliferate and metastasize.^5^ Therefore, to understand the specific mechanisms of proliferation, invasion, migration, and apoptosis of cisplatin‐resistant bladder cancer cells can help identify new therapeutic targets for this disease.

Rho GTPases are crucial regulators of the actin cytoskeleton, controling numerous cellular activities.^6^ These enzymes are essential in regulating the malignancy and metastatic dissemination of cancer cells.^7^ RAC3 is a member of Rho GTPases. Unlike in normal tissues, Rho GTPases are aberrantly overexpressed in different systemic tumours. They can boost the growth and drug resistance of cancer cells.^8^, ^9^, ^10^ In addition, their mutations in the cancer genome may affect the therapeutic efficacy of anticancer drugs, contributing to drug resistance.^11^ Meanwhile, aberrant DNA methylation has an important role in tumour development, which means it may have potential in the diagnosis and prognosis of cancer.^12^

Our previous study found an abnormally higher expression of RAC3 in tissues of bladder cancer than in paraneoplastic tissues, which may be related to tumour immune infiltration.^13^ However, RAC3 affects biological behaviours in cisplatin‐resistant bladder cancer cells and its specific regulatory mechanisms remain unknown.

JNK belongs to the mitogen‐activated protein kinase (MAPK) family and reportedly involves in many cellular functions. Activated JNK (p‐JNK) promotes the progression of colon, breast, and prostate cancers.^14^ A JNK‐specific inhibitor has been shown to block the of IL33 in colorectal cancer cell sphere formation.^15^ Inhibition of JNK also increases the sensitivity of hepatocellular carcinoma to cisplatin treatment.^16^ RAC3 is highly expressed in chemotherapy‐resistant bladder cancer cells.^17^ Mira et al.^18^ found that RAC3 can independently and continuously activate JNK, whereas inactivated RAC3N17 subtantially blocked JNK activity, indicating that RAC3 may be located upstream of JNK. This study sought to determine whether RAC3 could affect the different biological behaviours of cisplatin‐resistant bladder cancer cells through the JNK pathway.

In this study, RAC3 analysed by a series of bioinformatics methods was found highly expressed in bladder cancer, which was associated with poor patient prognosis. RAC3 has good predictive ability for the survival of patients with bladder cancer. We also explored its gene mutation, methylation alteration of RAC3 in bladder cancer. Cancer stem cells can contribute to poor prognosis by promoting tumour progression, recurrence and development of resistance to anticancer drugs.^19^ In addition, RAC3 correlated with immune cell infiltration and cancer stemness. Fuethermore, we found that RAC3 showed a positive correlation with stemness index of cancer, which indicated the poor prognoses of patients caused by high‐level expression of RAC3. Bladder cancer cell lines and constructed cisplatin‐resistant ones were used in this study. RAC3 was found overexpressed in cisplatin‐resistant bladder cancer cells. Further studies have shown that RAC3 increases the proliferation and invasion ability of drug‐resistant cancer cells through the JNK/MAPK pathway. Last but not least, this study systematically investigated the role of RAC3 in cisplatin‐resistant bladder cancer cells. The findings of this study imply that RAC3 might serve as a novel therapeutic target for cisplatin‐resistant bladder cancer.

## 2 MATERIALS AND METHODS

### 2.1 Online dataset analysis

Cancer‐associated RNA sequences in BLCA and clinicopathological and survival data were downloaded from the UCSC Xena network (https://xenabrowser.net/datapages/) for 424 cases, which included 405 cancer patients and 19 normal controls. The expression profiles of four bladder cancer‐related genes were obtained from the GEO database (https://www.ncbi.nlm.nih.gov/geo/), namely GSE13507, GSE3167, GSE37815 and GSE7476. R (version 4.0.5) was used to analyse the datasets. The criteria for screening differential genes were |logFC| >2 and *p*_adj_ = 0.05. The DESeq2, survival, survminer, and limma R packages were applied for the analysis. The Human Protein Atlas (HPA) database (https://www.proteinatlas.org/) provided data on RAC3 protein expression.

The single‐cell dataset GSE135337 contains seven tumour tissue samples of BLCA from the GEO database. Data were processed using Seurat, an R package. Quality control was performed according to the following criteria: (1) 1000–5000 expressed genes; and (2) less than 3% mitochondria‐associated genes. The harmony algorithm was adopted to remove the batch effect between different samples. A total of 2000 highly variable genes identified by the FindVariableFeatures function in the Seurat package. Cells were clustered by the FindClusters function (resolution = 0.5). We then annotated different cell types using the SingleR package and used HumanPrimaryCellAtlasData as a reference database to infer cell types. Finally, the expression levels of RAC3 in different cells were analysed.

### 2.2 Clinical prognosis analysis

COX regression analysis was performed and forest plots were drawn using the “survival” R package. Time‐dependent ROC curves (time ROC) were plotted using the “timeROC” R package to assess the predictive ability of RAC3 for survival of bladder cancer patients. Clinicopathologic and survival data of BLCA were downloaded from the UCSX Xena network, a nomogram including the expression of RAC3 was constructed, and calibration curves were plotted to assess the predictive value of the nomogram.

### 2.3 Genomic alterations of RAC3 in bladder cancer

Characterization of genomic alterations of RAC3 in the TCGA bladder cancer dataset was obtained from the cBioPortal database (https://www.cbioportal.org). Information on the types of mutation and mutation sites of RAC3 in bladder cancer was analysed.

### 2.4 Shiny Methylation Analysis Resource Tool (SMART)

Shiny Methylation Analysis Resource Tool (SMART, http://www.bioinfo‐zs.com/smartapp/) is used to analyse DNA methylation for the TCGA project. The correlation between RAC3 DNA methylation levels and its expression were examined with the tool. It was also used to compare the methylation of RAC3 gene in BLCA and normal tissue.

### 2.5 Association between RAC3 and immune infiltration and cancer stemness

The Estimation of Stromal and Immune cells in Malignant Tumour (ESTIMATE) algorithm estimates stromal and immune scores based on profiles of specific gene expression in stromal and immune cells. Based on the TCGA database, the effects of different expression levels of RAC3 on stromal scores, immune scores and ESTIMATE scores in bladder cancer was analysed to unveil their relationships with RAC3. The relationship between RAC3 and immune infiltration in bladder cancer was visualized using different R packages (“cibersort”). The stemness scores of patients with different tumours were obtained from the UCSC database, including Epigenetically regulated RNA expression‐based stemness index (EREG.EXPss) and mRNA expression‐based stemness index (RNAss). The correlation between RAC3 and stemness index was calculated using Spearman statistics.

### 2.6 Cell culture and materials

Cell lines from bladder cancer (T24, 5637 and BIU‐87) and cisplatin‐resistant bladder cancer cells from humans (BIU‐87‐DDP; ShcmBio, Shanghai, China) were used in this study. T24 cells were cultivated in McCoy's 5A medium, which contains 10% fetal bovine serum (FBS, DQ) and 1% penicillin. The 5637, BIU‐87 and BIU‐87‐DDP cells grew in 1640 medium (VivaCell) with 10% FBS, DQ, and 1% penicillin. The cells were cultured at 37°C under 5% CO_2_ and in sterile conditions. SP600125 (MedChemExpress, HY‐12041, New Jersey, USA) or anisomycin (MedChemExpress, HY‐18982, New Jersey, USA) were added to the culture medium to inhibit or activate the JNK/MAPK signalling pathway, respectively. Cells were treated with SP600125 at a concentration of 10 μM for 12 h or with anismycin at a concentration of 1 μM for 12 h.

### 2.7 Cell transfection and reagents

To abtain RAC3 overexpression, the cDNA of RAC3 was amplified and cloned into the pcDNA3.1 (GENERAL BIOL, Anhui, China) vector. RAC3 knockdown plasmid siRAC3 was constructed by Biotech Bioengineering (Shanghai, China). The pcDNA3.1 vector and plasmids were transfected into bladder cancer cells using the HighGene plus Transfection reagent (ABclonal, Hubei, China) according to the manufacturer's instructions as follows. Take 4 μg of plasmid and add it to a centrifuge tube containing 200 μL of serum‐free DMEM basal medium and mix well. Then add 8 μL of HighGene transfection reagent and mix well. Add 200 μL of plasmid/HighGene transfection reagent complex to the wells of a 6‐well cell culture plate. Gently shake the cell culture plate for even distribution. After 4–6 h of cell transfection, replace half the volume with fresh complete medium. Cells were collected when they had attained approximately 80% confluence. Proteins were isolated for Western blotting to quantify the degree of expression of RAC3, which was used to determine the effect of RAC3 transfection.

### 2.8 Western blot

Proteins were extracted using the RIPA protein lysate (Solarbio, R0010, Beijing, China), and the concentration of protein was measured using the BCA kit (Solarbio, PC0020, Beijing, China) and then corrected. The separation gel was set up according to the target protein's molecular weight. Equal amounts of protein (20 μg) were separated through 10% SDS‐PAGE and transferred to PVDF membranes (Solarbio, YA1701, Beijing, China). Then, 5% skim milk was used for blocking the membranes, followed by overnight incubation with the primary antibodies at 4°C. The samples were then treated for 2 h with the matching secondary antibodies. The samples were visualized using the ECL luminescent solution for colour development. The results were quantified using ImageJ software to analyse the bands. Beta‐actin (mouse, HuaBio, EM21002, Hangzhou, China) was used as an internal reference. The primary antibodies used included: RAC3 (rabbit, Abcam, ab124943, Cambridge, MA, United States), JNK (rabbit, HuaBio, ET1601‐28, Hangzhou, China), and p‐JNK (rabbit, HuaBio, ET1609‐42, Hangzhou, China). The secondary antibodies included anti‐rabbit IgG (Bioss, K008, Beijing, China) and anti‐mouse IgG (Bioss, K009, Beijing, China). The experiment was repeated three times for each subgroup.

### 2.9 CCK‐8 assay

The CCK‐8 kit (Solarbio, CA1210, Beijing, China) was used to assess cell viability and determine the effects of RAC3 and the JNK/MAPK signalling pathways on the proliferation of bladder cancer cells. Next, a cell suspension at the desity of 5 × 10^4^ cells/mL was inoculated into 96‐well plates for 24, 48 and 72 h preculture (37°C, 5% CO_2_), and 10 μL CCK‐8 solution was added to each of the 96‐well plates on the next day followed by cell incubation for 2 h. A microplate reader was used to measure the absorbance at 450 nm for the 96‐well plates. The experiment was repeated six times for each subgroup.

### 2.10 Transwell invasion and migration assays

To measure the amounts of cell migration and invasion, transwell assays were performed. Transwell chambers (BIOFIL, TCS‐003‐02, Guangzhou, China) were placed in 24‐well plates. The cells were digested with 0.05% trypsin–EDTA and resuspended. The cell concentration (5 × 10^5^ cells/well) was adjusted to inoculate into the upper chamber, where the matrix‐free gel was employed to detect the level of cell migration. The chambers coated with the matrix gel (Solarbio, M8370, Beijing, China) were used to detect the level of cell invasion. Serum‐free DMEM medium was introduced to the upper chamber while 500 μL of DMEM medium containing 20% FBS was introduced to the lower chamber for induction and both chambers were incubated for 24 h. A cotton swab was used to remove the cells from the upper chamber's surface after incubation. The cells underneath the membrane were fixed with 4% paraformaldehyde and stained for 5–10 min with 0.2% crystal violet. The migrating or invading cells were examined under a microscope with findings quantified using ImageJ software. The experiment was repeated six times for each subgroup.

### 2.11 Wound‐healing assay

In 6‐well plates, 5 × 10^5^ cells were seeded for growth. When the density of cultured cells reached 90%, a 20‐μL pipette tip was used to wipe down the midline of each well to create scratches of uniform width. The cells were then cultivated at 37°C and 5% CO_2_ for 24 h and observed under a microscope, with images collected separately. The mean gap length was calculated using ImageJ software to analyse the level of cell migration. The healing rate was calculated as follows: healing rate = (initial trace area—final trace area)/initial trace area × 100%. The experiment was repeated three times for each subgroup.

### 2.12 Flow cytometry‐based apoptosis detection

An FITC Annexin V Apoptosis Detection Kit I (BD, 556547, New Jersey, USA) was used to detecte apoptosis. Cells were digested with EDTA‐free trypsin and resuspended. Annexin V‐FITC was added before incubation for 1 h, followed by another 5 min after adding propidium iodide (PI). Samples were analysed using a flow cytometer (BD Biosciences, CA, USA).

### 2.13 Analysis of RAC3‐related genes and their construction of protein networks in bladder cancer

The 100 genes associated with RAC3 in bladder cancer were analysed in the LinkedOmics database (http://www.linkedomics.org/), including 50 positively associated genes and 50 negatively associated genes. We selected the top 50 genes most related to RAC3 and uploaded them to the STRING database (https://cn.string‐db.org/) for the construction of protein–protein interaction (PPI) networks to observe potential protein interactions.

### 2.14 Statistical analysis

All outcomes were collected by conducting three or six independent replicate experiments and were presented as mean ± SD. The statistical software R (version 4.0.5), SPSS 22.0 and GraphPad Prism 9 were used to examin the experimental data. The “survivor” R package was employed to perform the Kaplan–Meier survival analysis. Independent samples *t*‐test and ANOVA were used for the analysis of statistics from two or more experimental groups. At *p* < 0.05, differences were deemed statistically significant.

## 3 RESULTS

### 3.1 RAC3 was differentially expressed in bladder cancer tissues compared to normal tissues

**FIGURE 1:**
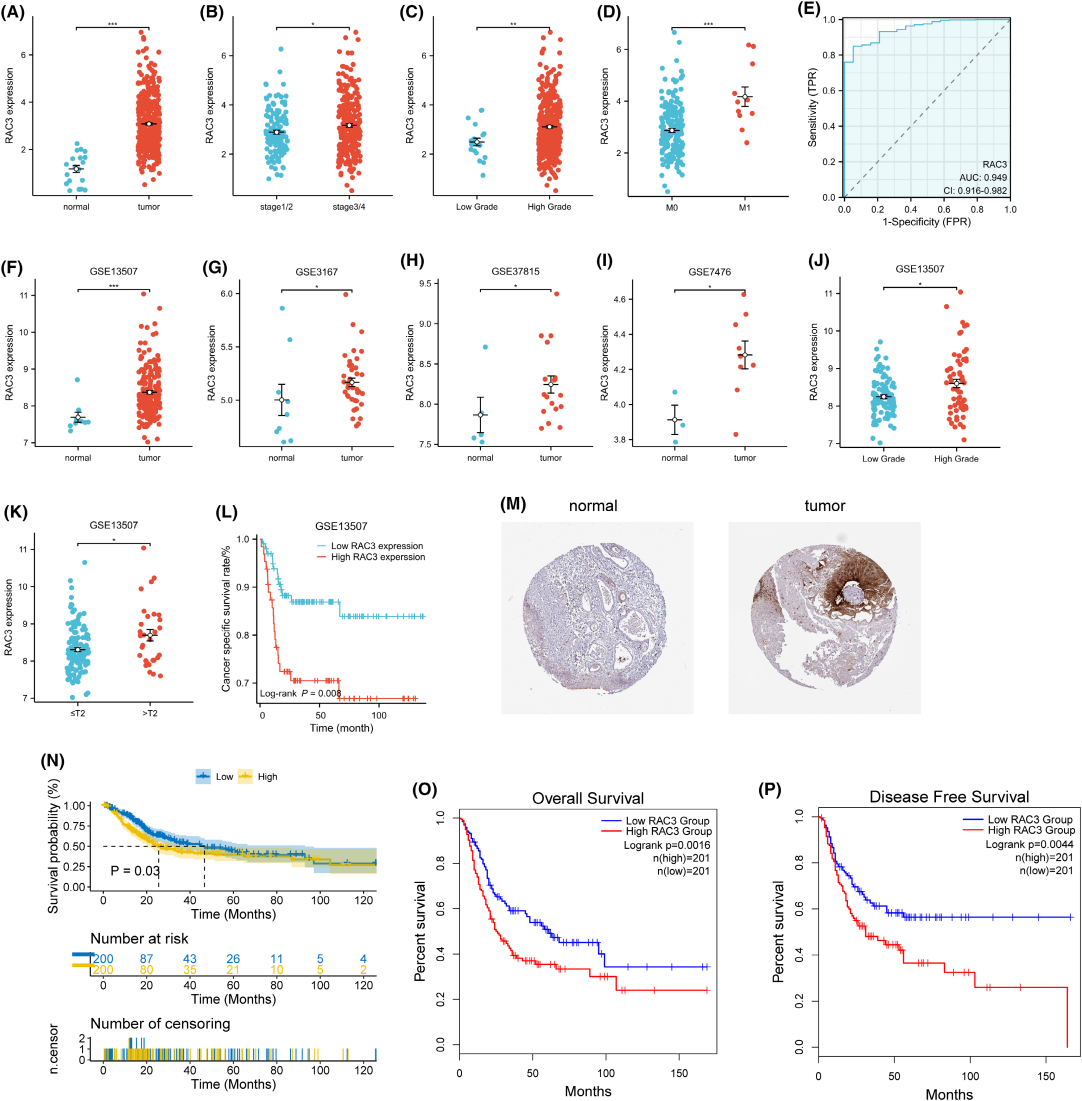
The upregulation of RAC3 corresponds to poor patient prognosis. (A) RAC3 expression differs between bladder cancer tissues and normal tissues. (B) RAC3 expression differs across the stages of bladder cancer. (C) RAC3 expression differs across the grades of bladder cancer. (D) RAC3 expression differs across stages of bladder cancer in patients with distant tumour metastases. (E) ROC curves for RAC3 in bladder cancer. (F) The expression of RAC3 in the GSE13507 dataset. (G) RAC3 expression in the GSE3167 dataset. (H) RAC3 expression in the GSE37815 dataset. (I) RAC3 expression in the GSE7476 dataset. (J) In the GSE13507 dataset, RAC3 expression differed in different grades of bladder cancer. (K) In the GSE13507 dataset, RAC3 expression differed in different T‐stage bladder cancers. (L) Relationship between RAC3 expression levels and cancer‐specific survival in the GSE13507 dataset. (M) RAC3 protein expression in normal bladder tissue and uroepithelial carcinoma. (N) Kaplan–Meier analysis was applied to analyse the survival curves of RAC3 expression variations in patients with bladder cancer. (O, P) The relationship between RAC3 expression and overall survival (OS). (P) The relationship between RAC3 expression and disease‐free survival (DFS). The data is presented as the mean ± SD. *p < 0.05, **p < 0.01, ***p < 0.001.

The mRNA expression of RAC3 in bladder cancer and healthy tissues from BLCA, downloaded from the UCSC Xena network, was determined by searching The Cancer Gene Atlas (TGCA). Relevant clinicopathological and survival data of bladder cancer patients were also obtained from TGCA. The comparative analysis revealed that RAC3 was highly expressed in samples of bladder cancer compared with healthy samples (Figure 1A). RAC3 was highly expressed in tissues of high‐grade and late‐stage bladder cancer (Figure 1B,C) and tissues of patients with distant metastases (Figure 1D). The excellent diagnostic ability of RAC3 for bladder cancer can be shown by constructing the ROC curve (Figure 1E). However, there was no significant difference in the expression level of RAC3 between T stage and N stage (Figure S1A,B). For grading and M‐staging of bladder cancer, RAC3 still has a good diagnostic ability (Figures S1C,D). To further verify differential expression of RAC3 in bladder cancer, we further collected four GEO datasets for analysis. The results revealed that RAC3 was highly expressed in tissues of bladder cancer (Figure 1F–I). The GSE13507 dataset was chosen for further analysis because it involves a higher number of patients of bladder cancer. The results showed that high level expression of RAC3 was associated with higher grading and T‐staging (Figure 1J,K). This result was consistent with those found in the analysis of the TGCA data. The results also showed that high‐level expression of RAC3 was alinked to lower cancer‐specific survival (Figure. 1L). The overexpression of RAC3 protein in uroepithelial carcinoma was confirmed in the HPA database (Figure. 1M).

The Kaplan–Meier survival analysis was used to determine the prognostic significance of RAC3 which may cause significantly lower survival in patients with more prominent expression than in those with poor expression (Figure 1N). The association of RAC3 with low survival in patients with bladder cancer was also verified by analysing the GEPIA database (http://gepia.cancer‐pku.cn/; Figure 1O,P). The data showed that expression of RAC3 is at a high level in tissues of bladder cancer, which could lead to poor grading, poor staging, and metastatic spread. Moreover, the survival rate was lower in patients with high‐level expression of RAC3. These results indicate RAC3 may be a prognostic factor for patients with bladder cancer.

Some necessary clinical information of BLCA patients including age, gender, race, TNM stage, histologic grade, pathologic stage, OS event, DSS event and PFI event are listed in Table 1. We determined the correlation between the expression of RAC3 and clinicopathologic features of BLCA. The high‐level expression of RAC3 was associated with advanced M stage (*p* = 0.014), high‐histologic grade (*p* = 0.003), advanced pathologic stage (*p* = 0.045), smoking (*p* = 0.002), OS event (*p* = 0.047) and PFI event (*p* = 0.022).

**TABLE 1:** Relationship between RAC3 expression and clinicopathological features in bladder cancer tissue samples.

Characteristics	Low expression of RAC3	High expression of RAC3	*p* value
*n*	206	206	
Age, *n* (%)			
≤70	121 (58.7%)	111 (53.9%)	0.321
>70	85 (41.3%)	95 (46.1%)	
Gender, *n* (%)			
Female	54 (26.2%)	54 (26.2%)	1.000
Male	152 (73.8%)	152 (73.8%)	
Race, *n* (%)			
Asian	29 (14.7%)	15 (7.6%)	0.078
Black or African American	11 (5.6%)	12 (6.1%)	
White	157 (79.7%)	171 (86.4%)	
Pathologic T stage, *n* (%)			
T1&T2	66 (34.7%)	57 (30.3%)	0.359
T3&T4	124 (65.3%)	131 (69.7%)	
Pathologic N stage, *n* (%)			
N0	125 (67.2%)	113 (62.1%)	0.305
N2 & N3 & N1	61 (32.8%)	69 (37.9%)	
Pathologic M stage, *n* (%)			
M0	113 (98.3%)	88 (90.7%)	0.014
M1	2 (1.7%)	9 (9.3%)	
Histologic grade, *n* (%)			
Low grade	17 (8.3%)	4 (2%)	0.003
High grade	187 (91.7%)	201 (98%)	
Pathologic stage, *n* (%)			
Stage I & Stage II	76 (37.1%)	57 (27.8%)	0.045
Stage III & Stage IV	129 (62.9%)	148 (72.2%)	
Smoker, *n* (%)			
No	69 (34.3%)	40 (20.2%)	0.002
Yes	132 (65.7%)	158 (79.8%)	
OS event, *n* (%)			
Alive	125 (60.7%)	105 (51%)	0.047
Dead	81 (39.3%)	101 (49%)	
DSS event, *n* (%)			
No	145 (72.5%)	128 (64.6%)	0.091
Yes	55 (27.5%)	70 (35.4%)	
PFI event, *n* (%)			
No	128 (62.1%)	105 (51%)	0.022
Yes	78 (37.9%)	101 (49%)	

Abbreviations: DSS, disease‐specific survival; OS, overall survival; PFI, progression‐free interval.

**FIGURE 2:**
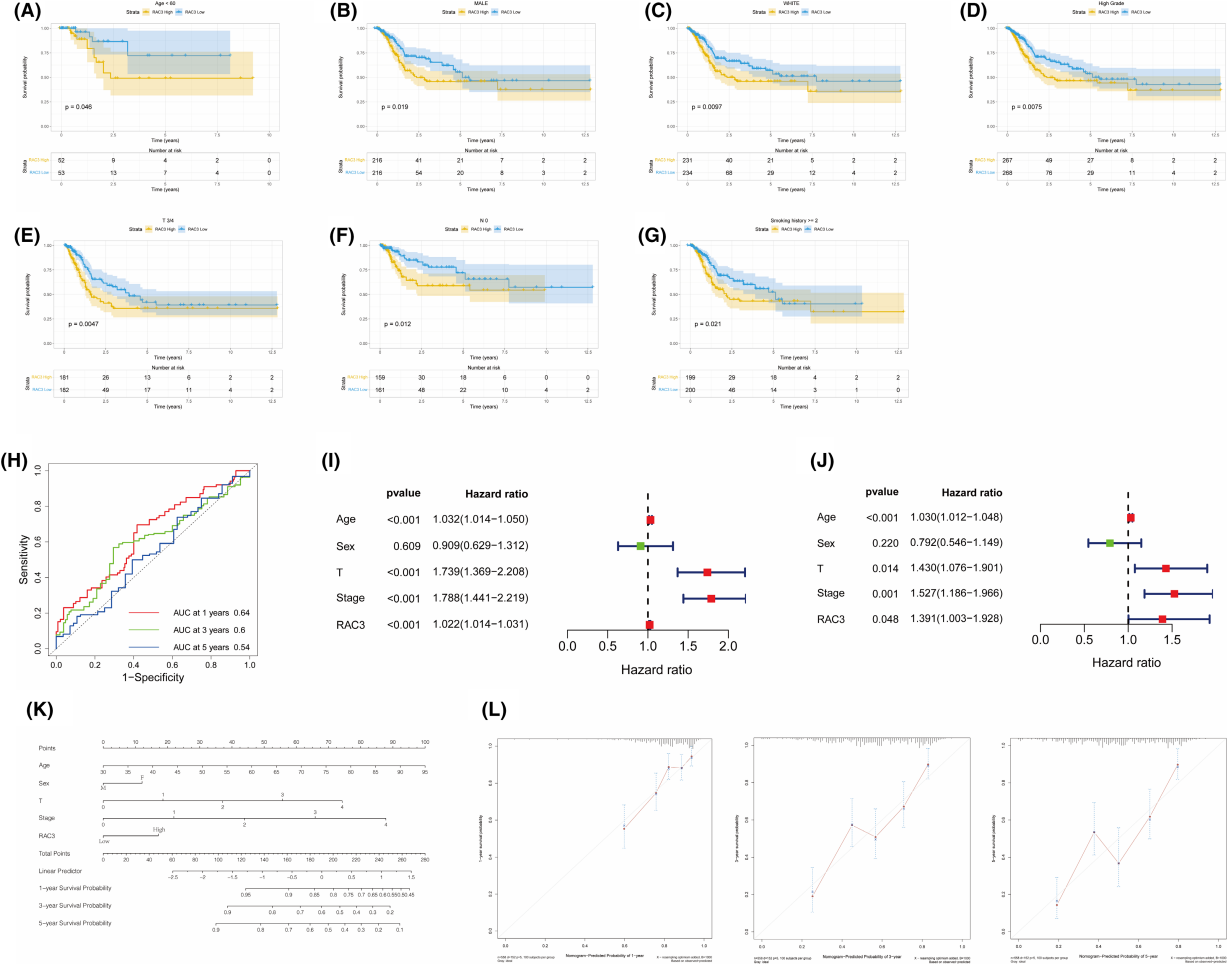
RAC3 as a predictor of bladder cancer. (A) Kaplan–Meier curves show survival differences between high and low RAC3 expression in patients <60 years of age. (B) Kaplan–Meier curves showing survival differences between high and low RAC3 expression in male patients. (C) Kaplan–Meier curves show survival differences between high and low RAC3 expression in white populations. (D) Kaplan–Meier curves show survival differences between high and low RAC3 expression in patients with high‐grade bladder cancer. (E) Kaplan–Meier curves showing survival differences between high and low RAC3 expression in patients with T3/4‐staged bladder cancer. (F) Kaplan–Meier curves showing survival differences between high and low RAC3 expression in patients with N0‐staged bladder cancer. (G) Kaplan–Meier curves showing survival differences between high and low RAC3 expression in bladder cancer patients with a smoking history of ≥2 years. (H) ROC curves for RAC3 in bladder cancer. (I, J) Univariate and multivariate COX regression analyses were performed to analyse the association between age, sex, T, stage and RAC3 expression and bladder cancer survival. (K) A nomogram was constructed using age, sex, T, stage, and high or low RAC3 expression (with median as the cutoff value). (L) Calibration curve for nomogram.

For the above analysis, we wonder whether RAC3 has a more significant impact on the prognosis of patients with bladder cancer in some subgroups. In subgroup survival analysis, we found that high level expression of RAC3 was associated with lower survival rates in patients aged <60 years, male, white population, high grade, T3/4 stage, N0 stage and smoking history ≥2 years (Figure 2A–G).

### 3.2 RAC3 as a biomarker for bladder cancer

We constructed ROC curves to assess the predictive ability of RAC3 in patients with bladder cancer (Figure 2H). The areas under the curve (AUCs) at 1, 3 and 5 years were 0.64, 0.6 and 0.54, respectively, which shows that RAC3 has a good predictive ability in predicting 1‐ and 3‐year survival for patients. However, its ability in predicting patients' 5‐year survival is poor. The univariate and multivariate COX regression analyses showed that age, T, stage and the expression of RAC3 were closely associated with the prognosis of patients with bladder cancer (Figure 2I,J). In addition, we further constructed a nomogram including RAC3 (Figure 2K), in order that it can be better applied to the clinic. A calibration curve was used to evaluate the accuracy of this nomogram (Figure 2L), which showed that the nomogram had a good predictive ability.

### 3.3 Genetic and epigenetic alterations of RAC3 in BLCA

**FIGURE 3:**
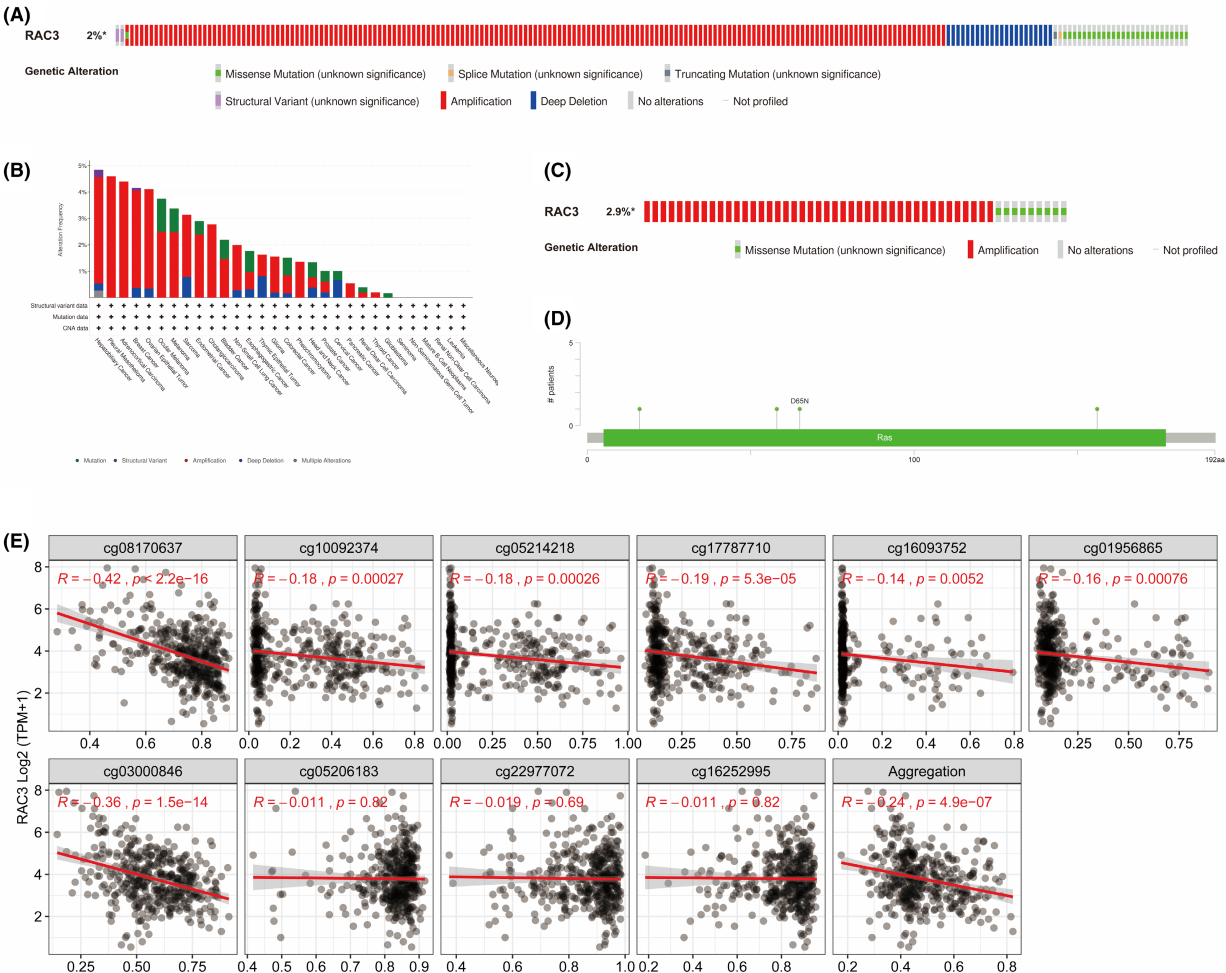
Genetic mutation and methylation alteration of RAC3 in BLCA. (A) RAC3 mutation frequency in pan‐cancer. (B) Major mutation types of RAC3 in pan‐cancers. (C) RAC3 mutation frequency in bladder cancer. (D) The mutation number and site of the RAC3 genetic alterations. (E) Correlation of RAC3 methylation levels and gene expression in bladder cancer patients.

We analysed the status of genetically altered RAC3 in different cancers in the cBioPortal database. The results showed that the mutation frequency of RAC3 in pan‐cancer was about 2%. The main types of genetic alterations were “amplification” and “deep deletion” (Figure 3A,B). The mutation frequency of RAC3 in bladder cancer was up to 2.9% with “amplification” as the main type (Figure 3C). In addition, the putative copy number alterations of RAC3 in Genomic Identification of Targets of Significance in Cancer (GISTIC) include many types, mainly “gain”, “diploid”, “amplification” and “shallow deletion” (Figure S2A). Furthermore, RAC3 was identified to have four mutation sites in the Ras region, located between amino acids 0 and 192. Among them, D65N is the most common mutation site. (Figure 3D).

In addition to DNA copy number variation, mRNA expression is also related to the degree of methylation of the gene. In this regard, we further analysed the methylation status of RAC3 in patients with bladder cancer using the SMART App. The results indicated the existence of seven methylation sites which were negatively correlated with the expression level of RAC3 (cg08170637, cg10092374, cg05214218, cg17787710, cg16093752, cg01956865 and cg03000846). What is more, the overall methylation of CpG sites was negatively correlated with the expression of RAC3 (Figure 3E). Among them, cg08170637 was negatively correlated with RAC3 expression, and the methylation level of cg08170637 in bladder cancer tissue was significantly reduced (Figure S2B). We further used TISIDB to analyse the impacts of the methylation level of RAC3 in BLCA on immune cell immunoinhibitor, immunostimulator, chemokines and chemokine receptors to explore their relationships with RAC3 (Figure S3). It is suggested that RAC3 methylation may play an important role in tumour immunity.

### 3.4 Single‐cell analysis of the expression of RAC3 in BLCA

**FIGURE 4:**
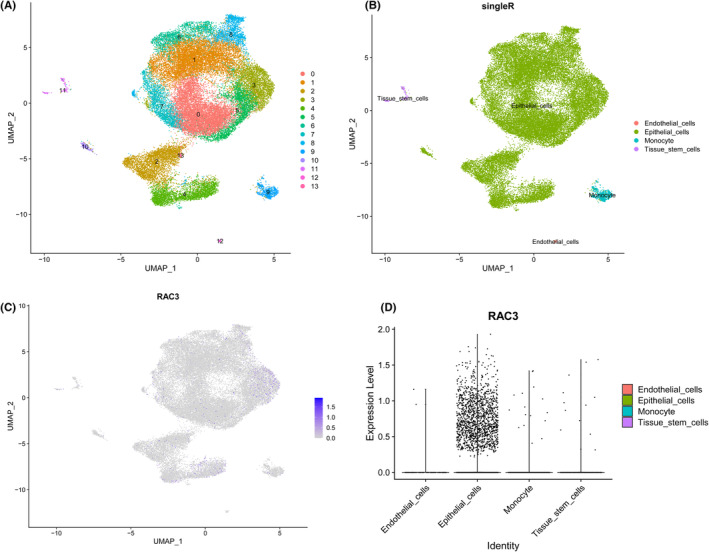
Detection of RAC3 expression by single cell analysis. (A) Cell clusters for GSE135337 of 7 BLCA patients. (B) Cell markers for clusters' annotation. (C, D) RAC3 expression in tissues of seven BLCA patients.

The above quality control standards (Figure S4A) were adopted to screen scRNA seq data. We set the resolution to 0.5 and identified 14 cell clusters (Figure 4A). Using the SingleR R package, 14 cell clusters were divided into four main types of cell, including endothelial_cells, epithelial_cells, monocytes, and tissue_stem cells (Figure 4B). As a biomarker of epithelial cells, we validated the expression of EPCAM in the “Epithelial_cells” subgroup and confirmed the accuracy of identification (Figure S4B). The results indicate that RAC3 is mainly expressed in epithelial cells (Figure 4C,D).

### 3.5 Relationship between RAC3 and immune cells and cancer stemness in bladder cancer

**FIGURE 5:**
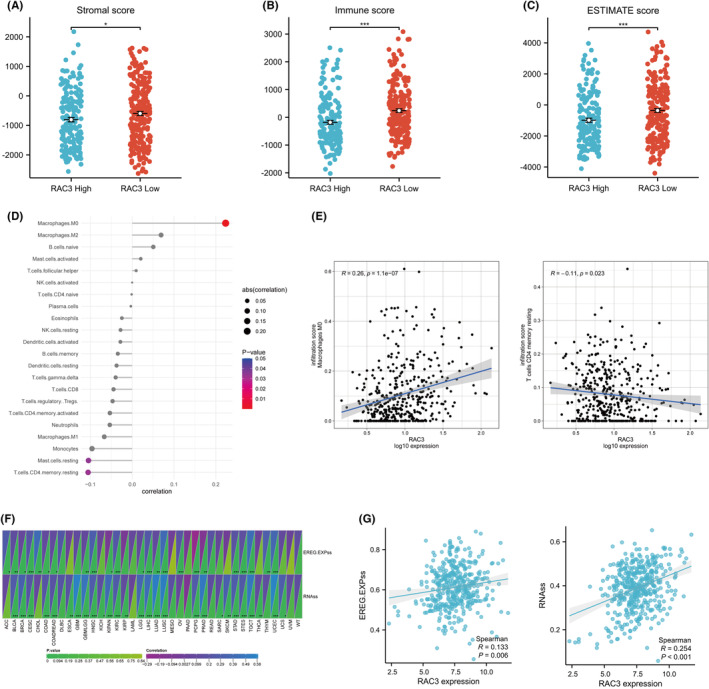
Association of RAC3 with immune infiltration and tumour stemness. (A–C)The score of stromal, immune, ESTIMATE in two groups (RAC3 high vs. RAC3 low). (D, E) Relationship between RAC3 and immune cell infiltration. (F, G) Relationship between RAC3 and tumour stemness score. *p < 0.05, **p < 0.01, ***p < 0.001.

In bladder cancer, we analysed the relationship between the expression of RAC3 and stromal score, immune score, and estimate score. It was found that the group with a high‐level expression of RAC3 had lower stromal score, immune score and estimate score (Figure 5A–C). Furthermore, we analysed the relationship between RAC3 and the level of immune cell infiltration (Figure 5D). RAC3 was found to be positively correlated with Macrophages M0 (*R* = 0.26, *p* < 0.01) while negatively correlated with T cells CD4 memory resting (*R* = −0.11, *p* < 0.05) (Figure 5E). In addition, we assessed the treatment scores for anti‐CTLA4 and anti‐PD1 inhibitors. A higher score for this treatment represented a better treatment effect. We found that the treatment score was higher in the group with a low‐level expression of RAC3 among all subgroups (Figure S5A), which suggests that anti‐CTLA4 therapy and anti‐PD1 therapy are better options for patients with a low‐level expression of RAC3. It also suggests that immunotherapy is less effective in patients with the overexpression of RAC3. Besides, our analysis revealed that patients with comparatively high IPS scores had a better prognosis (Figure S5C). What is more, the possible response of the expression of RAC3 to immunotherapy was assessed by analysing the TIDE score. The results showed that the group with a high‐level expression of RAC3 had a higher TIDE score. Meanwhile, high TIDE score was associated with poor prognosis in patients with bladder cancer (Figure S5B,C). The above suggests that a high‐level expression of RAC3 may have a worse outcome of immunotherapy. The stemness score was higher in tissues of bladder cancer than in normal tissues (Figure S6). In most tumours, there was a correlation between the expression level of RAC3 and the tumour stemness index (Figure 5F). Among them, in bladder cancer, the expression level of RAC3 was positively correlated with EREG.EXPss and RNAss (Figure 5G), which may indicate that a high level expression of RAC3 is associated with the development of drug resistance or the proliferation of tumour cell in bladder cancer cells.

### 3.6 RAC3 was prominently expressed in cisplatin‐resistant bladder cancer cells

**FIGURE 6:**
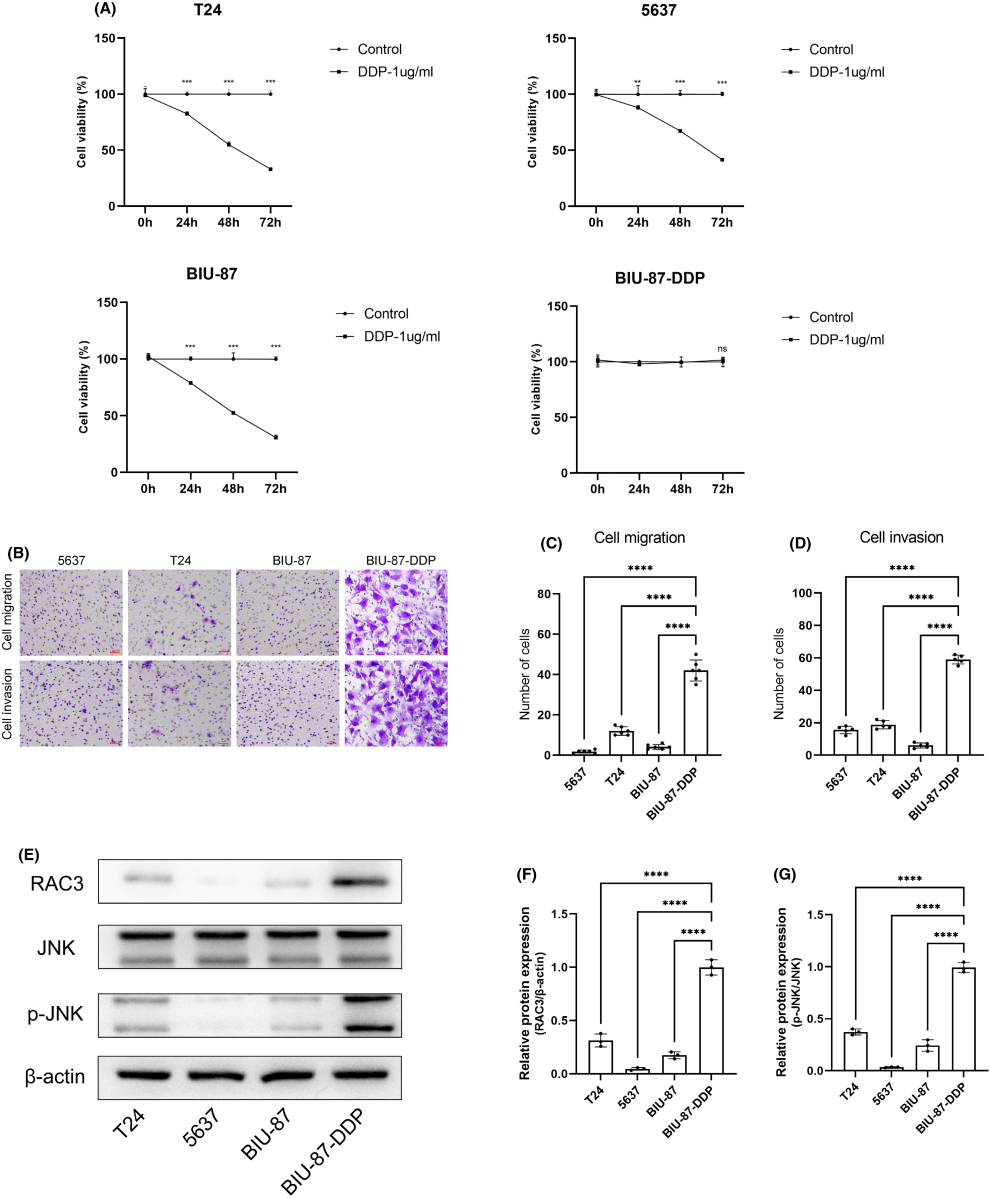
Cisplatin‐resistant bladder cancer cells. (A) A CCK‐8 assay was used to determine the cell viability of various bladder cancer cells following cisplatin therapy. (B–D) A transwell assay was used to evaluate bladder cancer cell migration and invasion capacity after cisplatin therapy. (E–G) Western blotting assay was performed to detect RAC3, JNK, and p‐JNK levels in T24, 5637, BIU‐87 and BIU‐87‐DDP cells. **p < 0.01, ***p < 0.001, ****p < 0.0001; ns, no significance.

Different bladder cancer cell lines and cisplatin‐resistant bladder cancer cell lines were treated with 1 μg/mL cisplatin for 24, 48 and 72 h. CCK‐8 and transwell assays were used to identify the proliferation and invasion of several cell lines following cisplatin therapy. The levels of proliferation and invasion in common bladder cancer cell lines (T24, 5637 and BIU‐87) decreased significantly after cisplatin treatment, suggesting there were significant differences after the treatment. Whereas, no significant difference was detected in the results of the cisplatin‐resistant bladder cancer cell line (BIU‐87‐DDP) after cisplatin treatment, indicating the development of drug resistance in bladder cancer cells was successful (Figure 6A–D). Western blotting was performed to determine the expression levels of RAC3 in the cisplatin‐resistant cell lines and three normal bladder cancer cell lines, whose results showed the expression levels of RAC3 were considerably higher in the cisplatin‐resistant bladder cancer cells than in the normal ones(Figure 6E,F).

### 3.7 The expression level of JNK in cisplatin‐resistant bladder cancer cells

In previous studies of tumours, RAC3 was found to be associated with cisplatin resistance^16^ and it activates JNK independently in breast cancer cells.^18^ This study sought to determine the association between RAC3 and the JNK signalling pathway in cisplatin‐resistant bladder cancer cells. Western blotting was used to identify proteins of JNK/MAPK pathway. RAC3 was overexpressed in the cisplatin‐resistant bladder cancer cells (BIU‐87‐DDP) compared with the normal ones (T24, 5637 and BIU‐87; Figure 6E,F). Besides, p‐JNK was also highly expressed in BIU‐87‐DDP (Figure 6E,G), while total JNK levels were not significantly different. The expression levels of RAC3 and p‐JNK were very likely to increase once cisplatin resistance was developed in the bladder cancer cells, indicating that RAC3 may be associated with the JNK/MAPK pathway.

### 3.8 RAC3 was effectively silenced and overexpressed

**FIGURE 7:**
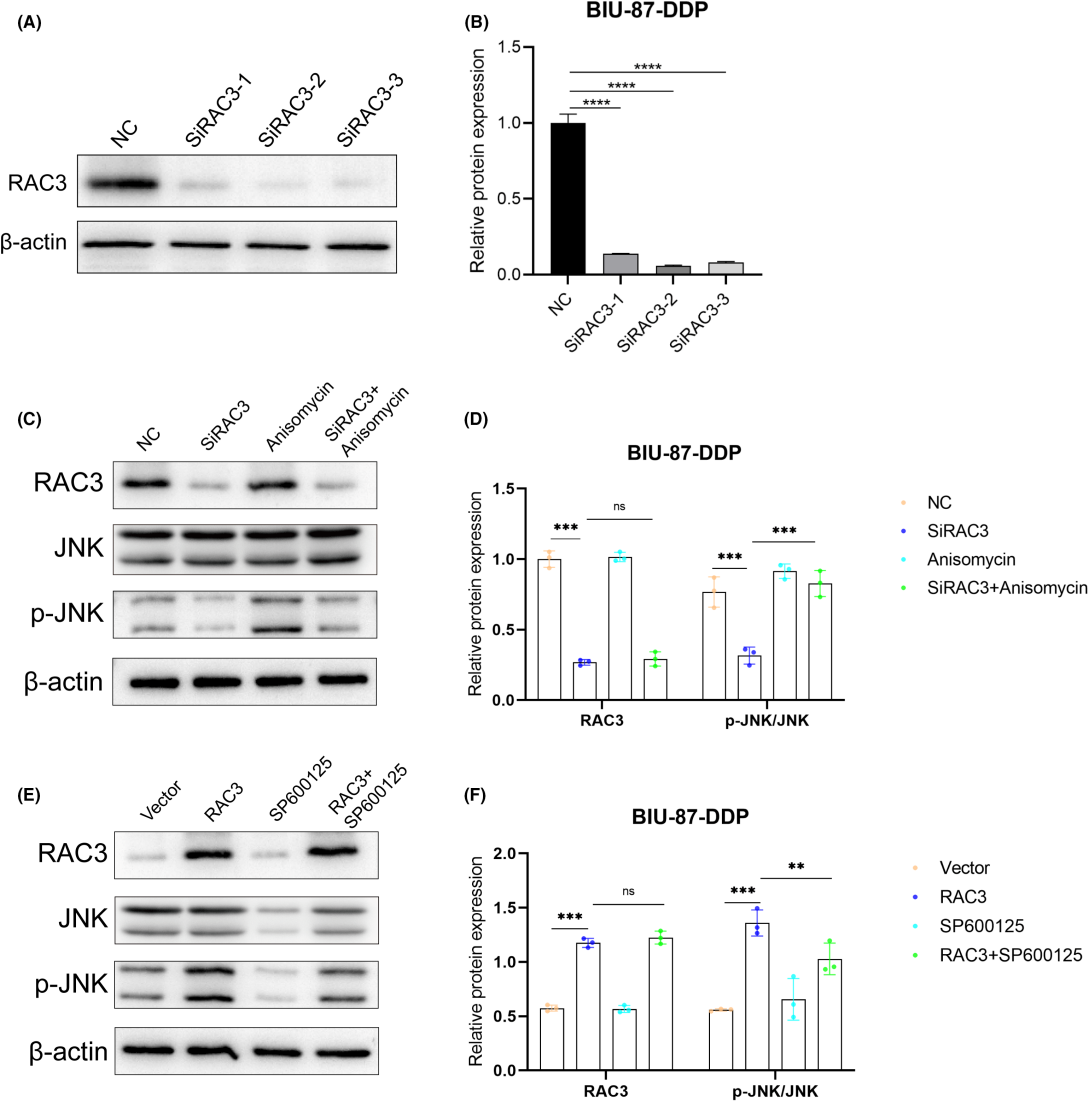
Effective silencing and overexpression of RAC3 in bladder cancer cells altered the JNK signalling pathway. (A–B) The effect of transfection of different siRNA sequences on RAC3 expression in BIU‐87‐DDP cells. (C–D) RAC3, JNK, and p‐JNK expression levels were detected by Western blotting after RAC3 silencing and treated with the JNK activator. (E–F) RAC3, JNK, and p‐JNK expression levels after RAC3 overexpression and after using JNK inhibitors, as detected by Western blotting. **p < 0.01, ***p < 0.001, ****p < 0.0001; ns, no significance.

Three siRAC3‐downregulated interference sequences were screened through Western blotting, and they could significantly downregulate the protein expression of RAC3. Among them, the siRAC3‐2 sequence exhibited the highest transfection efficiency and was therefore selected for the subsequent study (Figure 7A,B). The overexpressed or silenced RAC3 plasmid was transfected into BIU‐87‐DDP cells. The outcomes of western blotting showed the expression levels of RAC3 were significantly lower than in the control group after transfection with the siRAC3 plasmid (Figure 7C,D). Conversely, the levels were much higher after transfection with the overexpressed RAC3 (Figure 7E,F). The silencing and overexpression by transfection was confirmed to be successful based on what is been observed on the expression levels of RAC3.

### 3.9 RAC3 regulated the activity of the JNK/MAPK pathway

To learn more about the mechanism of RAC3 affecting the behaviour of cisplatin‐resistant bladder cancer cells, western blotting was used to detect the expression levels of proteins related to the JNK/‐MAPK pathway after BIU‐87‐DDP cells were transfected with different RAC3 plasmids. The knockout of RAC3 did not dramatically lower the expression of JNK, but it did considerably reduce its phosphorylation (Figure 7C,D). Similarly, the overexpression of RAC3 did not significantly affect the total expression of JNK, but the level of its phosphorylation increased (Figure 7E,F). These results indicate that in cisplatin‐resistant bladder cancer cells, RAC3 regulates the JNK/MAPK pathway by affecting the phosphorylation of JNK.

### 3.10 Silencing of RAC3 inhibited the proliferation of cisplatin‐resistant bladder cancer cells, while its overexpression promoted their proliferation

**FIGURE 8:**
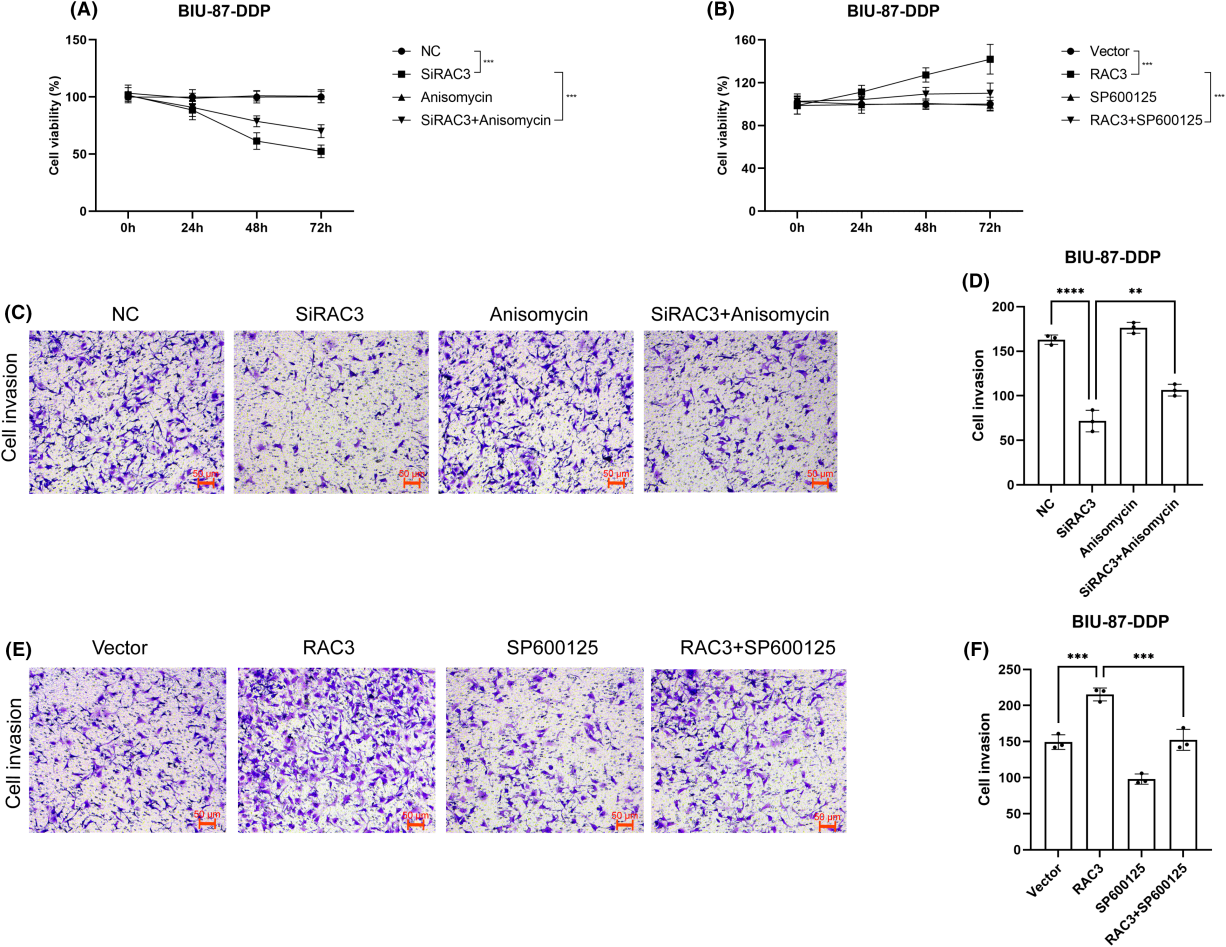
RAC3 activates JNK/MAPK signalling to enhance the proliferation and invasion of cisplatin‐resistant bladder cancer cells. (A) A CCK‐8 assay was used to assess the proliferative capacity of cells after RAC3 knockdown and transfection with the JNK activator anisomycin. (B) A CCK‐8 assay was performed to assess the proliferation capacity of cells after RAC3 overexpression and treated with the JNK inhibitor SP600125. (C, D) Transwell assay and quantification of the invasive capacity of cells after RAC3 knockdown and treated with the JNK activator anisomycin. (E, F) Transwell assay and quantification of invasive ability of cells after RAC3 overexpression and after using the JNK inhibitor SP600125. **p < 0.01, ***p < 0.001, ****p < 0.0001.

**FIGURE 9:**
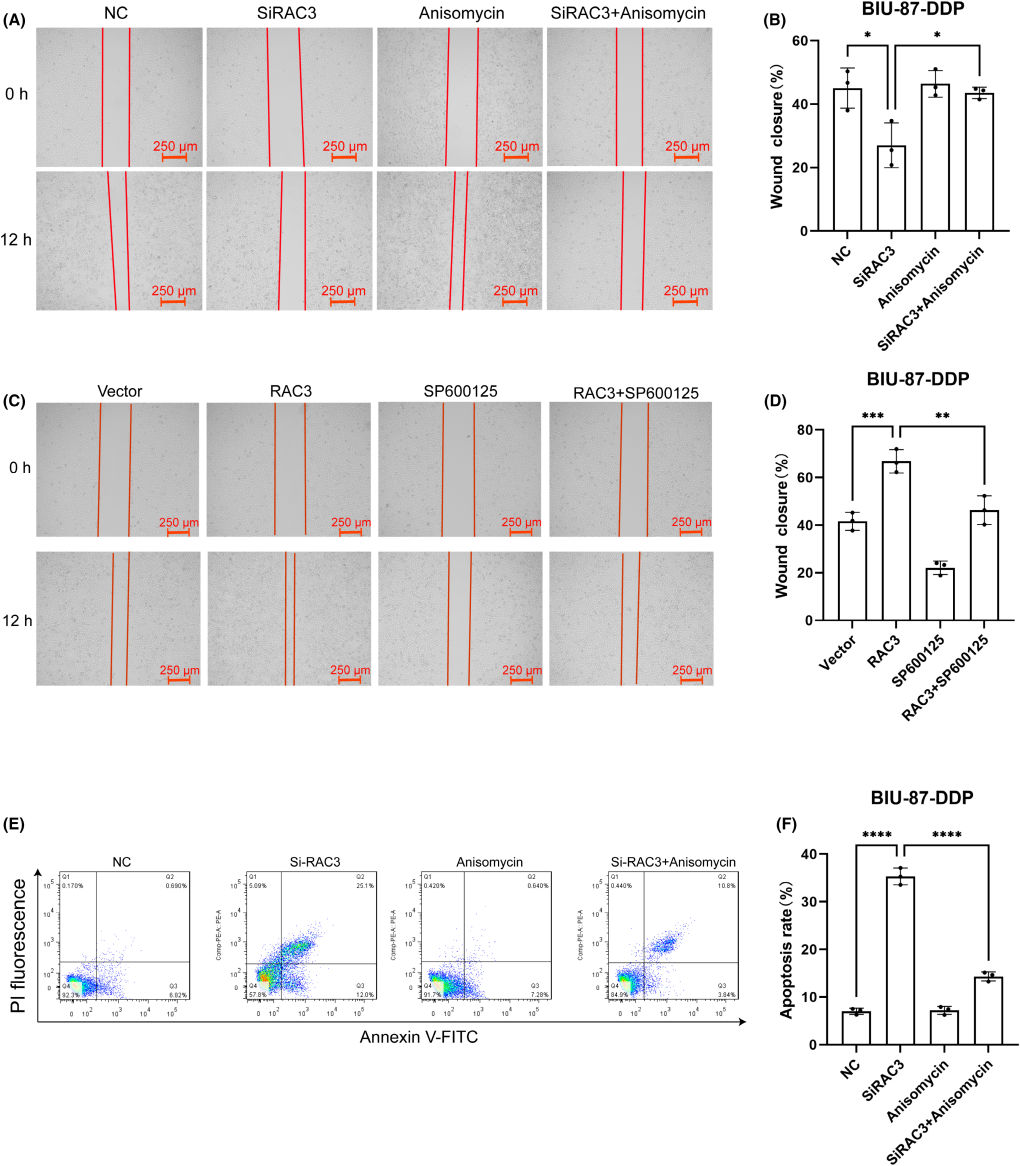
RAC3 activates JNK/MAPK signalling to promote the migration of cisplatin‐resistant bladder cancer cells. (A, B) A wound healing assay was performed to assess the migration capacity of cells after. RAC3 silencing and treatment with the JNK activator anisomycin. (C, D) Wound healing assay to assess the migration capacity of cells after RAC3 overexpression and treatment with the JNK inhibitor SP600125. (E, F) Flow cytometry assay of the apoptotic ratio of cells after RAC3 silencing and treatment with the JNK activator anisomycin. *p < 0.05, **p < 0.01, ***p < 0.001, ****p < 0.0001.

The CCK‐8 assay revealed that silencing of RAC3 reduced BIU‐87‐DDP cell proliferation (Figure 8A). Transwell and wound healing assays similarly revealed that silencing of RAC3 inhibited the migration and invasion capacity of BIU‐87‐DDP cells (Figures 8C,D and 9A,B). The apoptosis of cisplatin‐resistant bladder cancer cells after the silencing of RAC3 was examined using flow cytometry, and the results showed the percentage of apoptosis increased after RAC3 was silenced (Figure 9E,F). The abilities for proliferation, migration, and invasion of cells increased after BIU‐87‐DDP cells were transfected with overexpressed RAC3 plasmid (Figure 8B,E,F; Figure 9C,D). Silencing of RAC3 inhibited the proliferation, migration, and invasion of cisplatin‐resistant bladder cancer cells in vitro, while elevating the apoptosis of cancer cells in vitro. On the contrary, cells with overexpressed RAC3 exhibited increased proliferation, migration, and invasion.

### 3.11 RAC3 affected the proliferation, migration, invasion, and apoptosis of cisplatin‐resistant bladder cancer cells via the JNK/MAPK pathway

To study whether RAC3 influences the behaviours of cisplatin‐resistant bladder cancer cells via the JNK/MAPK pathway. We used the activator of JNK/MAPK signalling pathway (anisomycin) to treat cancer cells whose expression of RAC3 had already been knocked down. The results showed a considerable increase in the expression and in the phosphorylation level of JNK after treatment with the pathway‐specific activator (Figure 7C,D). After the overexpression of RAC3, BIU‐87‐DDP cells were further treated with the inhibitor of JNK/MAPK signalling pathway (SP600125). The results indicated a significant decrease in the expression and the phosphorylation level of JNK after treatment with the pathway‐specific inhibitor (Figure 7E,F), indicating the pathway activators and inhibitors affected the expression of JNK. Next, CCK‐8 and wound healing assays were performed to determine whether RAC3 affects various behaviours of cisplatin‐resistant bladder cancer cells by regulating the JNK/MAPK pathway. The activator treatment of the pathway significantly enhanced the proliferation, migration, and invasion of RAC3‐silenced BIU‐87‐DDP cells, and to some extent, reversed the inhibition of tumour progression triggered by the silencing of RAC3 (Figures 8A,C,D and 9A,B). Moreover, the proportion of apoptotic cells were reduced by activating this pathway (Figure 9E,F). Conversely, the abilities for proliferation, migration, and invasion of cancer cells (Figure 8B,E,F) were significantly weakened after the addition of the pathway inhibitor to the RAC3 overexpressed BIU‐87‐DDP cells (Figure 9C,D).

### 3.12 Exploring the expression of RAC3 in BLCA for gene clusters with positive and negatively correlation

**FIGURE 10:**
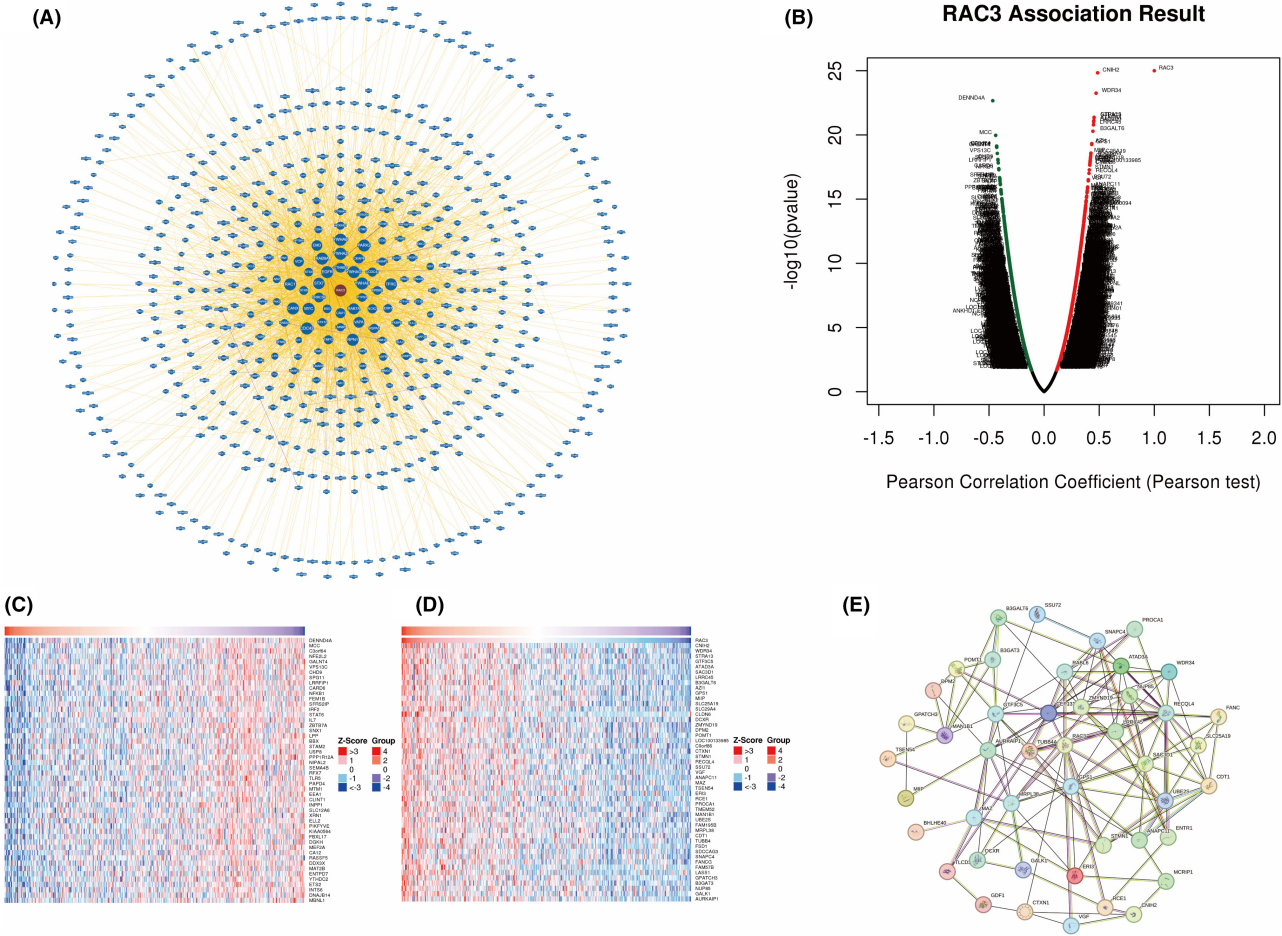
Analysis of RAC3 related genes. (A) BioGRID web platform was used to get RAC3‐interacted molecules. (B) A volcano plot depicting some genes associated with the RAC3 expression in BLCA. (C) Heat map indicating the top 50 genes that were positively associated with the RAC3 expression in BLCA. (D) Heat map indicating the top 50 genes negatively associated with the RAC3 expression in BLCA. (E) RAC3 related interacting protein network obtained through STRING tool.

We collected 631 molecules from human that may interact with RAC3 through the BioGRID network service (https://thebiogrid.org/) (Figure 10A). We demonstrated that the gene clusters were highly associated with the expression of RAC3 in bladder urothelial carcinoma (BLCA) by volcano plot based on the analysis of LinkedOmics database (http://www.linkedomics.org/) (Figure 10B). The heat map identified the top 50 genes that were positively and negatively correlated with the expression of RAC3 in BLCA (Figure 10C,D). We further uploaded these genes to the string database (https://cn.string‐db.org/) to generate a protein–protein interaction (PPI) networks for visualization after deleting proteins with no interaction (Figure 10E).

## 4 DISCUSSION

Bladder cancer is among the most prevalent malignant tumours of the urinary system, and muscle‐invasive bladder cancer is particularly prone to spreading.^20^, ^21^ This study analysed the difference in the expression of RAC3 between bladder cancer tissues and normal tissues using bioinformatics. A significantly high‐level expression of RAC3 was observed in bladder cancer tissues, which often leads to a poor prognosis of bladder cancer patients. In addition, we analysed genetic and epigenetic alterations of RAC3 in BLCA and discovered the prominent amplification mutations of RAC3. What is more, aberrant DNA methylation has been recognized to have an important role in tumour development,^22^ and our results showed that the expression level of RAC3 in bladder cancer was negatively correlated with the methylation levels of the cg08170637, cg10092374, cg05214218, cg17787710, cg16093752, cg01956865 and cg03000846 sites. It is consistent with our findings that an increase in the DNA methylation often leads to a decrease in gene expression.^23^ The tumour immune microenvironment is known to have a significant impact on tumour development, cancer outcome and cancer prognosis,^24^ whose analysis suggested that the stromal score, immune score, and ESTIMATE composite score decreased in the groups with high expression level of RAC3. This may be associated with poor prognosis of patients with bladder cancer. On the other hand, the stemness index of cancer can assess the similarity between tumour cells and stem cells, and it can also indicate the degree of tumour dedifferentiation.^25^ Therefore, we analysed the relationship between RAC3 and the stemness index of cancer. We found that RAC3 has a correlation with the cancer stemness in bladder cancer, which may suggest that RAC3 is associated with the generation of drug resistance and further proliferation of cancer cells during bladder cancer treatment.^26^

Currently, cisplatin plays a dominate role in treatment for advanced bladder cancer.^27^, ^28^ Cisplatin is widely used as a chemotherapeutic agent oncologically. A large portion of patients with initially sensitive tumours develop cisplatin resistance, eventually leading to a poor prognosis.^29^, ^30^ As a member in the subfamily of Rho GTPases, RAC3 (note that it is distinguished from the steroid/nuclear receptor coactivator Rac3^31^) promotes tumour cell proliferation and resistance to antitumor drugs. The overexpression of RAC3 may be linked to the induction and maintenance of cancer stem cells in various systemic tumours.^8^, ^9^, ^32^ RAC3 is overexpressed in lung cancer, breast cancer, or other tumours.^8^, ^9^, ^10^, ^33^ It has been shown that RAC3 mediates autophagy in bladder cancer cells via the PI3K/AKT/mTOR pathway.^34^ RAC3 overexpression can also increase bladder cancer cell proliferation and invasion by activating the JAK/STAT signalling pathway through PYCR1 upregulation,^35^ affecting bladder cancer development. Many bioinformatic analyses and in vitro cell‐based assays have confirmed the presence of aberrant high‐level expression of RAC3 in bladder cancer and it is associated with poor patient prognosis.^13^, ^36^, ^37^ In summary, RAC3 likely plays a role as an oncogene in different cancers. However, current studies on RAC3 mostly focus on experiments in common bladder cancer cells. Its role in cisplatin‐resistant bladder cancer cells in regulating the proliferation, migration, invasion, and apoptosis has not been investigated. Although cisplatin is the basis in chemotherapy for bladder cancer, many bladder cancer patients develop clinical resistance to cisplatin, which leads to treatment failure. Therefore, this study revealed that RAC3 was overexpressed in cisplatin‐resistant bladder cancer cells compared to normal ones. We investigated the effects of RAC3 on the proliferation, migration, invasion, and apoptosis of cisplatin‐resistant bladder cancer cells and the underlying regulatory mechanisms of RAC3.

Numerous studies have shown that the JNK/MAPK pathway significantly affects the development of cancer cells in various tumours. Specific p‐JNK inhibition can considerably reduce the proliferation and migration of cancer cells such as lung adenocarcinoma, oesophageal squamous cell carcinoma, and colon cancer cells, and can greatly induce the apoptosis of some cancer cells.^38^, ^39^, ^40^, ^41^ By influencing the expression of many molecules, the JNK signalling pathway can increase the growth and metastasis of cancer cells, whereas JNK activation can also inhibit apoptosis by inducing autophagy in various cancers. In cancer treatment, the activation of JNK pathway can promote the expression of PD‐L1, thereby enhancing the immune escape of tumours. Also, the susceptibility of cancer cells to cisplatin can be increased by suppressing JNK.^16^ In bladder cancer, phosphorylated JNK was significantly reduced by CCDC34 knockdown and was found to suppress the migration of cancer cells and induce their apoptosis.^42^ This suggests that CCDC34 regulates the proliferation, migration, and apoptosis of bladder cancer cells by regulating the JNK/MAPK signalling pathway. Another study found that MEX3C, as an oncogene, could encourage the growth of bladder cancer by regulating lipid metabolism via the MAPK/JNK pathway.^43^ It has also revealed that the activation of the JNK/MAPK signalling pathway causes the resistance of lung adenocarcinoma to gefitinib.^44^ Other study discovered that the inhibitors of JNK and MAPK increased the susceptibility of head and neck squamous cell carcinoma to cetuximab.^45^ RAC3 can influence the progression of cancer cell in many tumours by controlling the JNK/MAPK pathway. However, the potential regulatory mechanisms underlying the relationship between RAC3 and cisplatin‐resistant bladder cancer cells are still unknown.

Differences in the expression of RAC3 between the normal and cisplatin‐resistant bladder cancer cells were examined through Western blotting. Different protein expression of JNK/MAPK signalling pathway were also assessed. Changes in the activation of JNK/MAPK pathway and the proliferation, migration, invasion, and apoptosis of cisplatin‐resistant bladder cancer cells were examined by the silencing or overexpressing of RAC3. A series of behavioural changes in the cisplatin‐resistant bladder cancer cells were observed by treating the cells with activators or inhibitors of JNK/MAPK signalling pathway. Anisomycin, a JNK/MAPK signalling pathway activator, significantly upregulated phosphorylated JNK,^46^, ^47^ whereas JNK phosphorylation was specifically inhibited by SP600125, a JNK/MAPK inhibitor.^48^, ^49^ Anisomycin also partially restored the abilities for the proliferation and invasion of cisplatin‐resistant bladder cancer cells even though they had diminished after RAC3 was silenced. SP600125 reduced the proliferation and invasion of the cancer cells after induced by the overexpression of RAC3.

A high expression level of RAC3 is obvious in paclitaxel‐resistant lung adenocarcinoma cells. Moreover, RAC3 could affect the paclitaxel resistance by activating the PI3K/AKT pathway.^50^ Chen et al.^17^ found that RAC3 was highly expressed in chemotherapy‐resistant bladder cancer tissues. These researchers further treated bladder cancer cells by knocking down or overexpressing RAC3 followed by cisplatin or gemcitabine. They found that the knockdown of RAC3 reduced the resistance of bladder cancer cells to chemotherapeutic drugs and increased apoptosis. In contrast, the overexpression of RAC3 increased the resistance of bladder cancer cells to chemotherapeutic drugs. The knockdown of RAC3 was found to decrease the phosphorylation levels of PAK1 and ERK1/2, but did not affect the levels of total PAK1 and ERK1/2, which suggests that in bladder cancer cells, RAC3 can promote resistance to cisplatin by lifting the phosphorylation levels of PAK1‐ERK1/2. Future studies should examine whether RAC3 could also affect the ability for proliferation or invasion of BIU‐87‐DDP cells by influencing the phosphorylation levels of the PAK1/JNK axis or whether RAC3 affects various behaviours of BIU‐87‐DDP cells through the PAK1‐ERK1/2 axis.

In this study, we comprehensively analysed the effects of RAC3 in bladder cancer. And further explored it in cisplatin‐resistant bladder cancer cells. RAC3 was highly expressed in cisplatin‐resistant bladder cancer cells and could regulate the proliferation, migration, invasion, and apoptosis of these cancer cells by activating the JNK/MAPK signalling pathway. These findings suggest RAC3 plays a crucial role in the proliferation and invasion of cisplatin resistant‐bladder cancer cells. In clinical practice, RAC3 may serve as a possible therapeutic target for patients with cisplatin‐resistant bladder cancer.

## 5 CONCLUSION

In conclusion, our study revealed that RAC3 is overexpressed in bladder cancer and is associated with a range of poor prognoses. Associated with the proliferation, migration, invasion, and apoptosis, RAC3 was highly expressed in cisplatin‐resistant bladder cancer cells. RAC3 was also found to regulate a series of behaviours of cisplatin‐resistant bladder cancer cells by activating the JNK/MAPK signalling pathway. The results of this study indicate that RAC3 may be a new target for the clinical treatment of patients with cisplatin‐resistant bladder cancer.

## AUTHOR CONTRIBUTIONS

**Haodong Li:** Conceptualization (equal); data curation (equal); formal analysis (equal); funding acquisition (equal); investigation (equal); methodology (equal); software (equal); validation (equal); visualization (equal); writing – original draft (equal). **Hongxuan Ma:** Conceptualization (equal); data curation (equal); formal analysis (equal); investigation (equal); writing – original draft (equal). **JianHua Ma:** Investigation (equal); methodology (equal); visualization (equal); writing – original draft (equal). **Fei Qin:** Data curation (equal); formal analysis (equal); software (equal). **Siqi Fan:** Investigation (equal); resources (equal); software (equal). **Shaopeng Kong:** Formal analysis (equal); software (equal). **Sitao Zhao:** Data curation (equal); formal analysis (equal); software (equal). **Jianguo Ma:** Methodology (equal); project administration (equal); visualization (equal); writing – original draft (equal); writing – review and editing (equal).

## FUNDING INFORMATION

This study was supported by the Hebei Provincial Excellent Talents in Clinical Medicine Project (2022180).

## CONFLICT OF INTEREST STATEMENT

The authors declare that they have no conflict of interest to disclose.

## CONSENT

This study does not involve informed consent.

## Supporting information


Figure S1.

Figure S2.

Figure S3.

Figure S4.

Figure S5.

Figure S6.

## Data Availability

The data that support the findings of this study are available from the corresponding author upon reasonable request.
