# Supplementary figures and images for: Unveiling the role of RAC3 in the growth and invasion of cisplatin‐resistant bladder cancer cells
Source: J Cell Mol Med. 2024 Jun 7;28(11):e18473. doi: 10.1111/jcmm.18473 (PMC11157678; doi:10.1111/jcmm.18473)

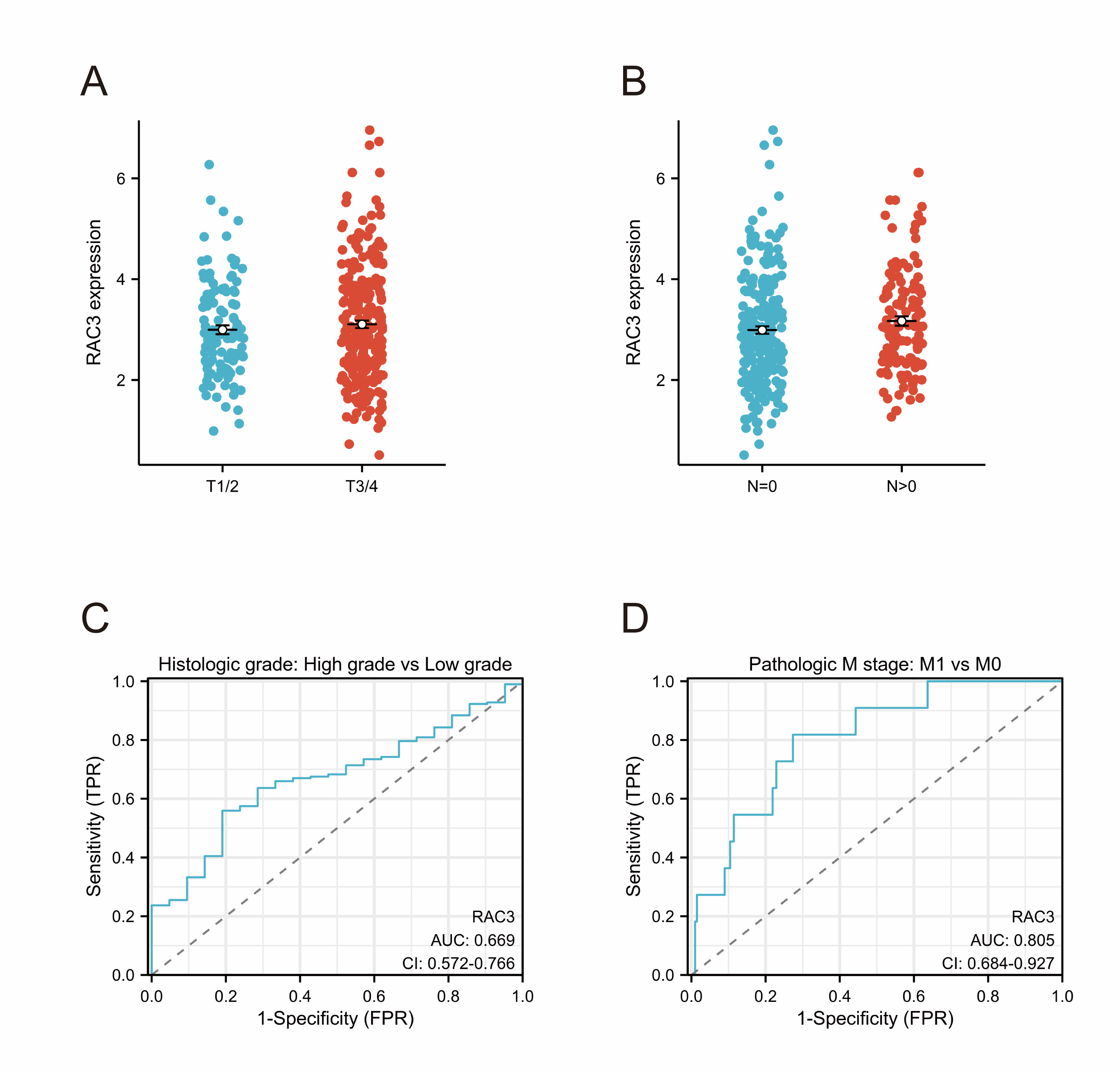

Supplement: Supplementary file 1 — Figure S1. [file JCMM-28-e18473-s001.tif]

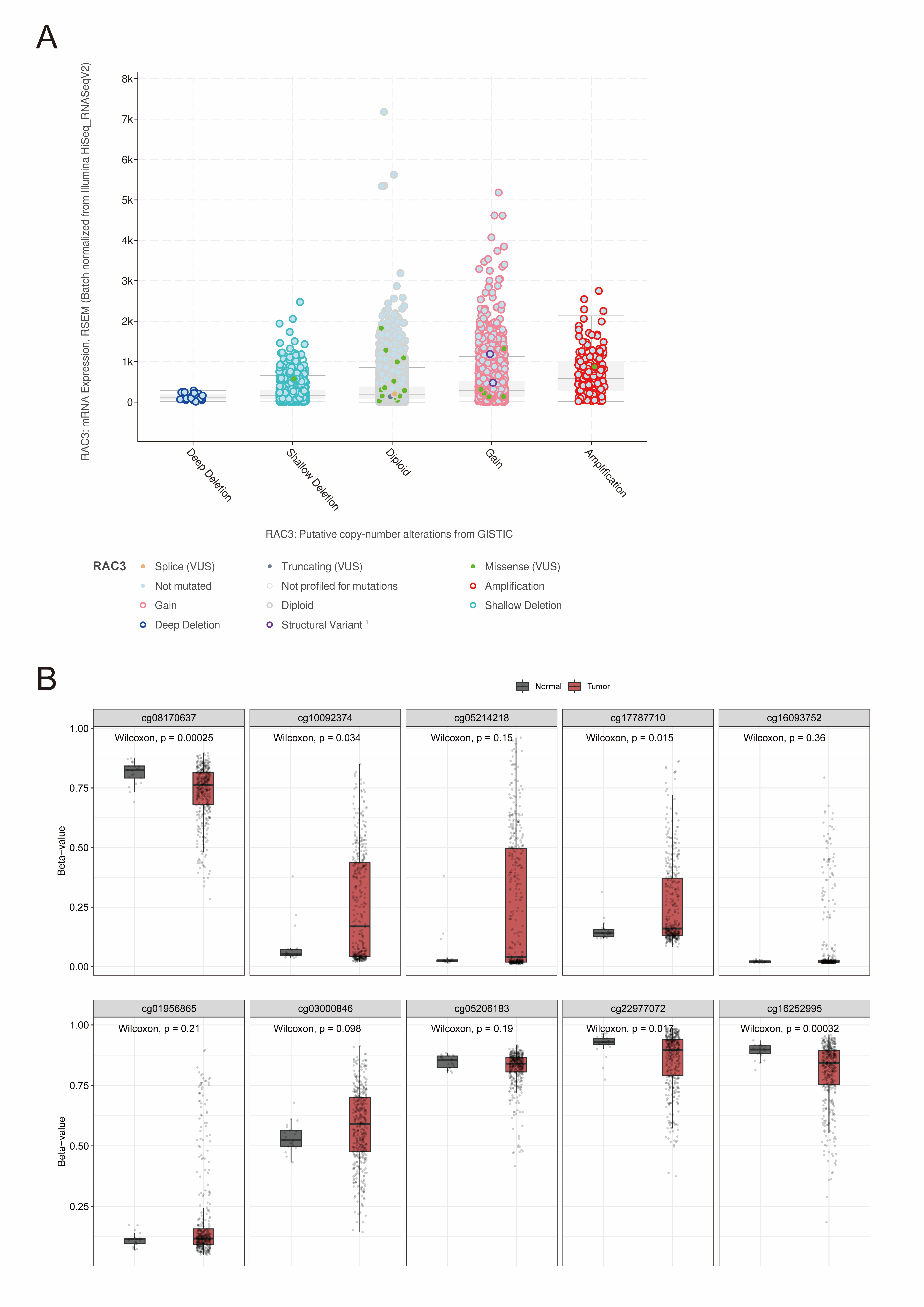

Supplement: Supplementary file 2 — Figure S2. [file JCMM-28-e18473-s002.tif]

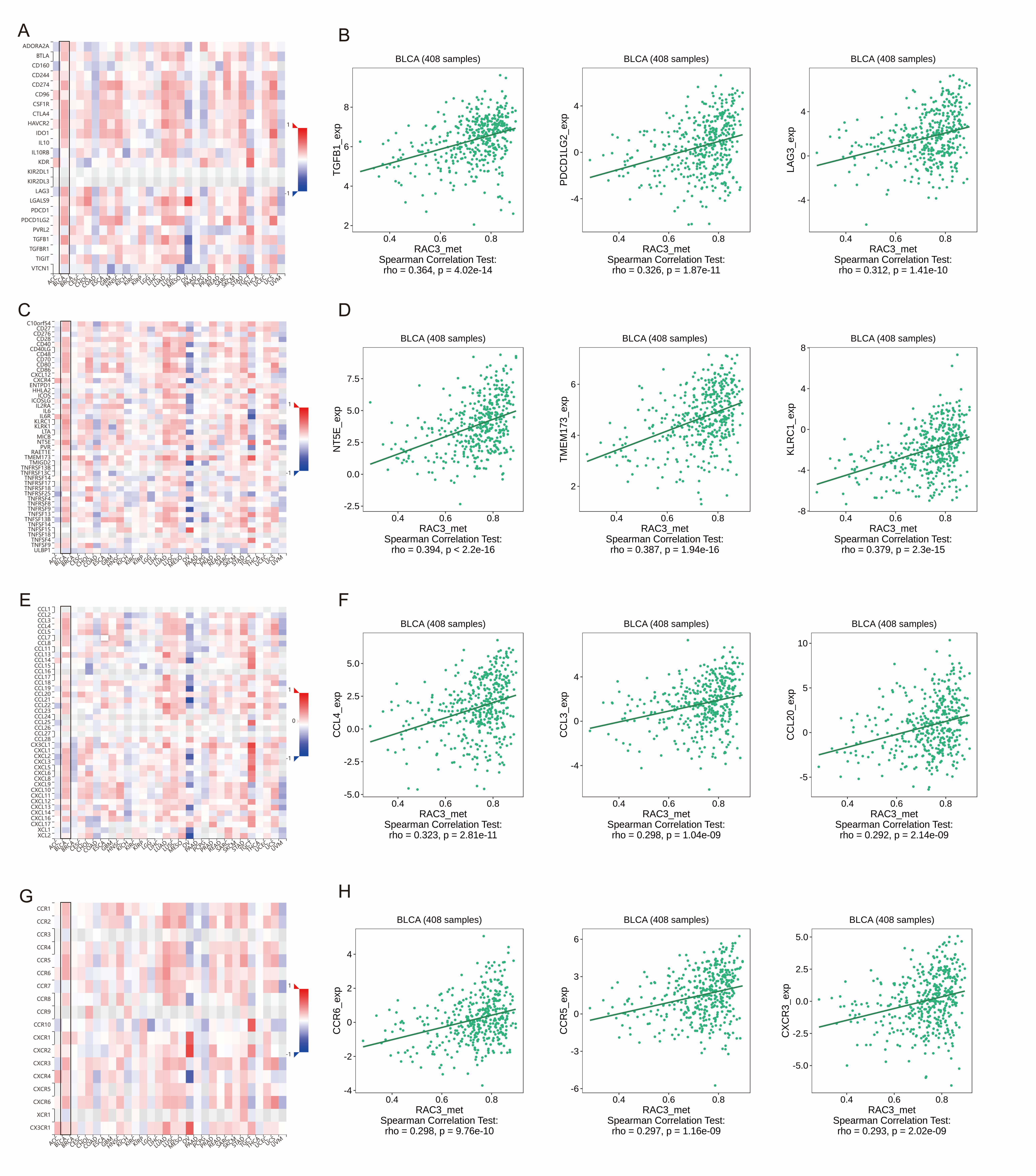

Supplement: Supplementary file 3 — Figure S3. [file JCMM-28-e18473-s004.tif]

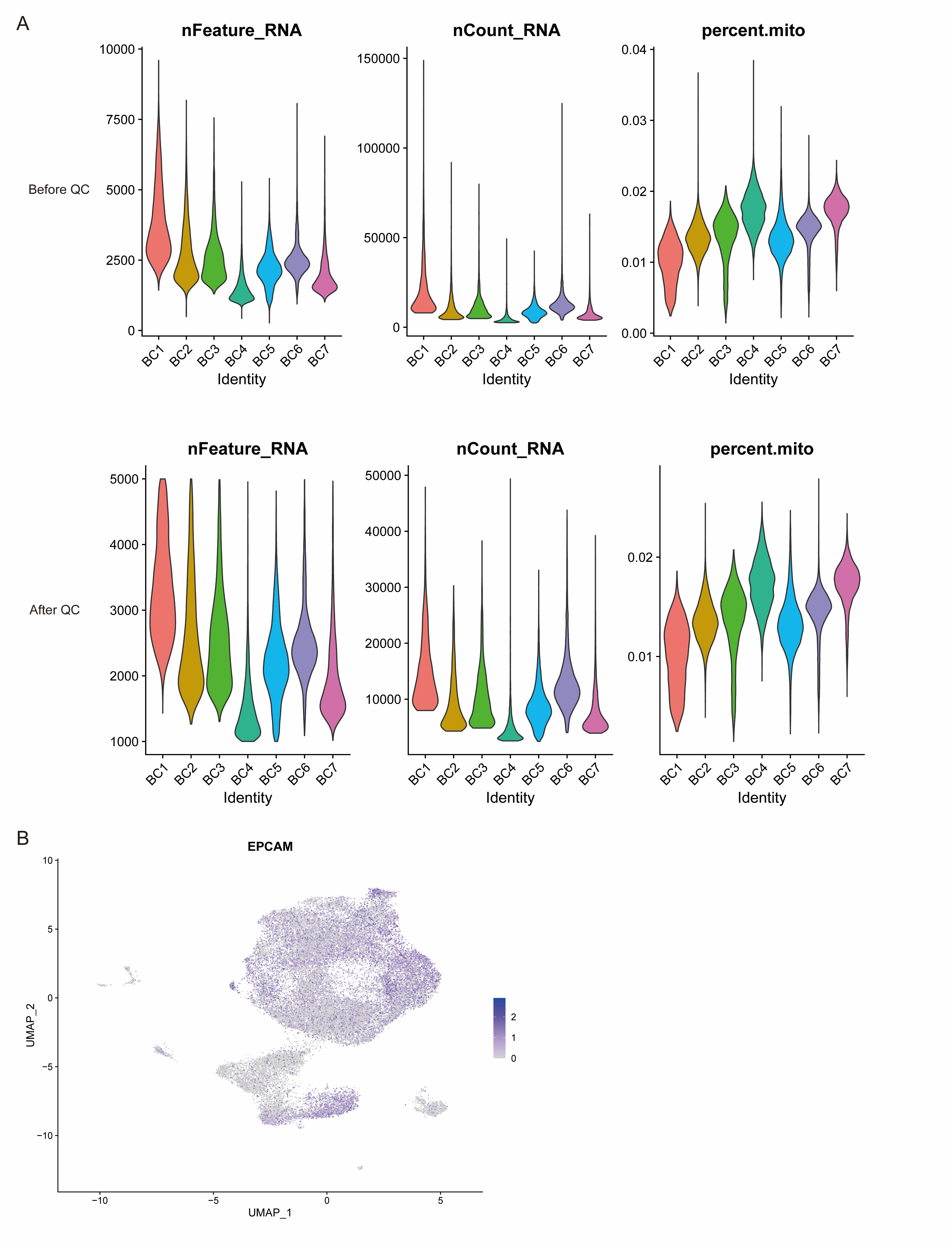

Supplement: Supplementary file 4 — Figure S4. [file JCMM-28-e18473-s003.tif]

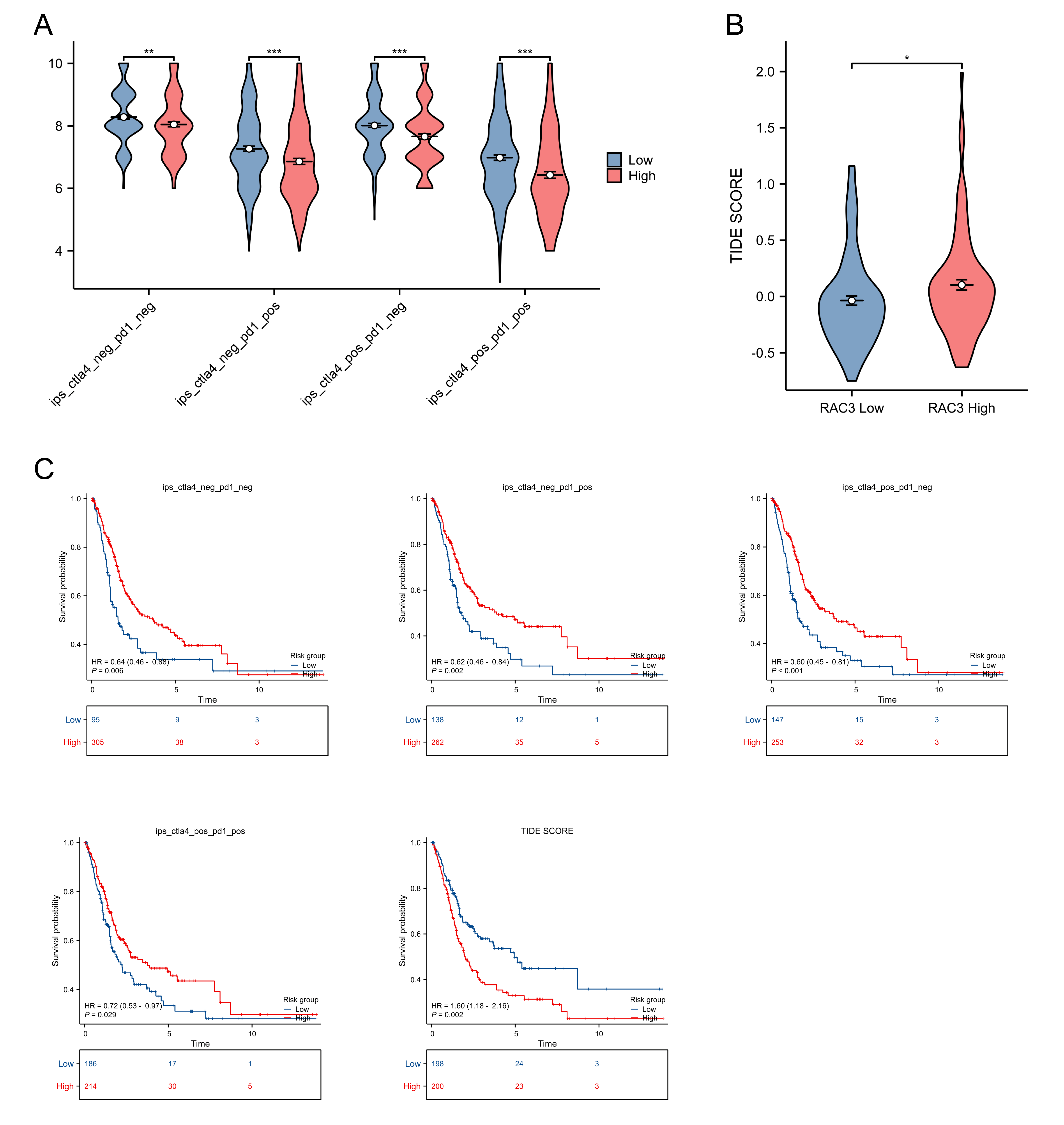

Supplement: Supplementary file 5 — Figure S5. [file JCMM-28-e18473-s005.tif]

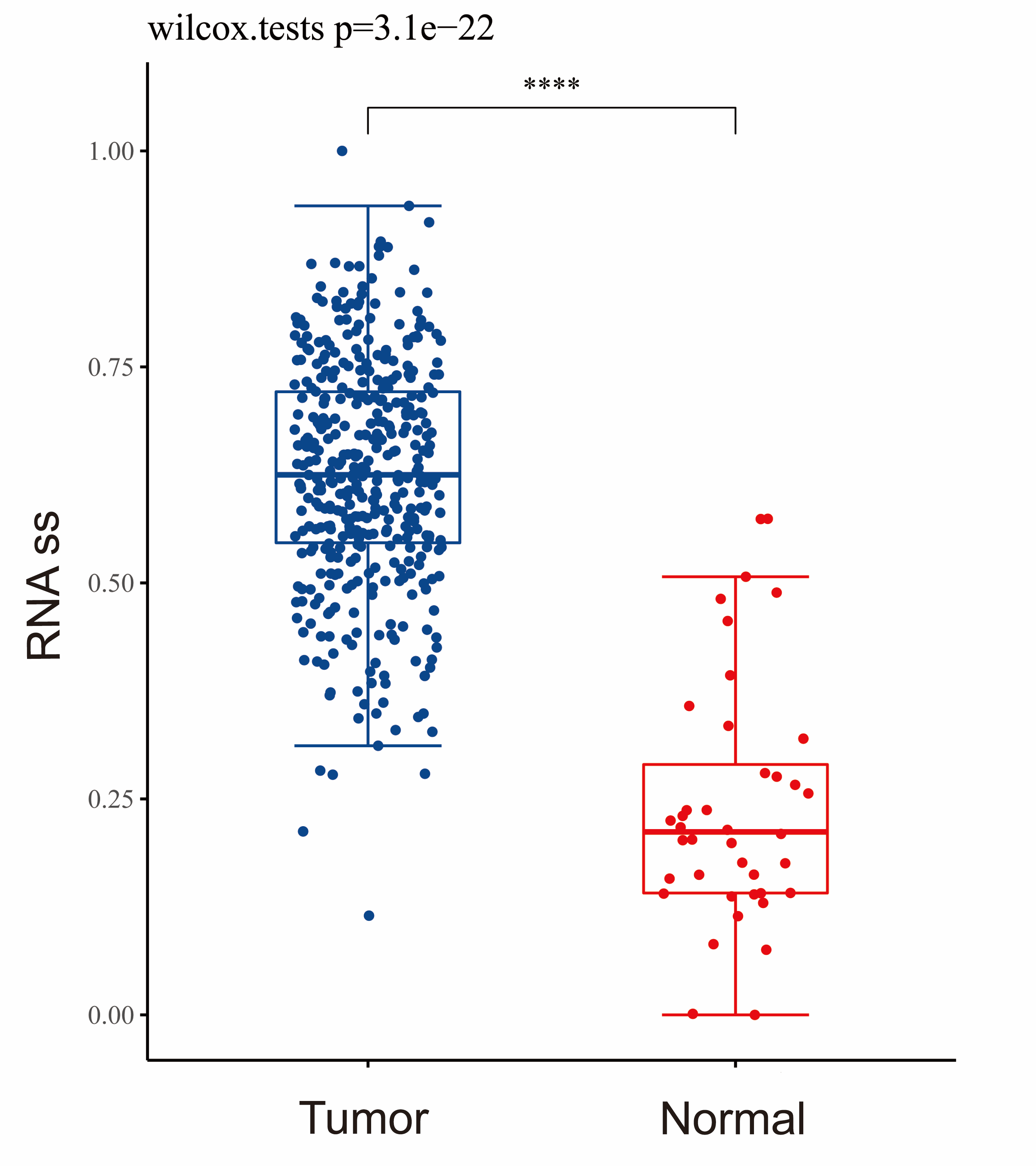

Supplement: Supplementary file 6 — Figure S6. [file JCMM-28-e18473-s006.tif]
